# *N*′-(2,5-Dihydroxy­benzyl­idene)-2-hydr­oxy-3-methyl­benzohydrazide

**DOI:** 10.1107/S1600536810013395

**Published:** 2010-04-17

**Authors:** You-Yue Han, Yong-Hong Li, Qiu-Rong Zhao

**Affiliations:** a Department of Chemistry and Life Science, Chuzhou University, Chuzhou, Anhui 239000, People’s Republic of China

## Abstract

In the title compound, C_15_H_14_N_2_O_4_, the dihedral angle between the two benzene rings is 4.1 (2)°. The mol­ecule adopts an *E* configuration with respect to the C=N bond. There are intra­molecular O—H⋯N and O—H⋯O hydrogen bonds in the mol­ecule. In the crystal structure, mol­ecules are linked through inter­molecular N—H⋯O and O—H⋯O hydrogen bonds, forming chains running along the *c* axis.

## Related literature

For the biological properties of hydrazone compounds, see: Patil *et al.* (2010 ▶); Cukurovali *et al.* (2006 ▶). For the crystal structures of hydrazones, see: Mohd Lair *et al.* (2009 ▶); Lin & Sang (2009 ▶); Suleiman Gwaram *et al.* (2010 ▶). For hydrazone compounds recently reported by us, see: Han & Zhao (2010*a* ▶,*b* ▶). For bond-length data, see: Allen *et al.* (1987 ▶). For similar compounds, see: Li & Ban (2009 ▶); Lo & Ng (2009 ▶); Ning & Xu (2009 ▶); Zhu *et al.* (2009 ▶).
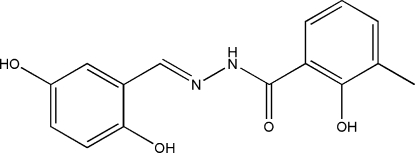


## Experimental

### 

#### Crystal data

C_15_H_14_N_2_O_4_
*M* *_r_* = 286.28
Monoclinic, 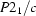

*a* = 13.333 (2) Å
*b* = 7.316 (1) Å
*c* = 13.738 (2) Å
β = 94.742 (2)°
*V* = 1335.5 (3) Å^3^
*Z* = 4
Mo *K*α radiation
μ = 0.11 mm^−1^
*T* = 298 K
0.25 × 0.23 × 0.22 mm

#### Data collection

Bruker SMART CCD area-detector diffractometer
Absorption correction: multi-scan (*SADABS*; Bruker, 2001 ▶) *T*_min_ = 0.974, *T*_max_ = 0.977
7636 measured reflections
3002 independent reflections
1457 reflections with *I* > 2σ(*I*)
*R*_int_ = 0.087

#### Refinement

*R*[*F* ^2^ > 2σ(*F* ^2^)] = 0.052
*wR*(*F* ^2^) = 0.136
*S* = 0.82
3002 reflections
197 parameters
1 restraint
H atoms treated by a mixture of independent and constrained refinement
Δρ_max_ = 0.22 e Å^−3^
Δρ_min_ = −0.26 e Å^−3^

### 

Data collection: *SMART* (Bruker, 2007 ▶); cell refinement: *SAINT* (Bruker, 2007 ▶); data reduction: *SAINT*; program(s) used to solve structure: *SHELXTL* (Sheldrick, 2008 ▶); program(s) used to refine structure: *SHELXTL*; molecular graphics: *SHELXTL*; software used to prepare material for publication: *SHELXTL*.

## Supplementary Material

Crystal structure: contains datablocks global, I. DOI: 10.1107/S1600536810013395/om2333sup1.cif

Structure factors: contains datablocks I. DOI: 10.1107/S1600536810013395/om2333Isup2.hkl

Additional supplementary materials:  crystallographic information; 3D view; checkCIF report

## Figures and Tables

**Table 1:** Hydrogen-bond geometry (Å, °)

*D*—H⋯*A*	*D*—H	H⋯*A*	*D*⋯*A*	*D*—H⋯*A*
O1—H1⋯O2	0.82	1.82	2.5452 (19)	147
O3—H3⋯N2	0.82	1.85	2.576 (2)	146
O4—H4⋯O3^i^	0.82	1.97	2.774 (2)	169
N1—H1*A*⋯O4^ii^	0.90 (2)	2.27 (2)	3.072 (2)	148 (2)

Symmetry codes: (i) 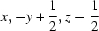; (ii) 
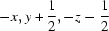
.

**Fig. 1.**
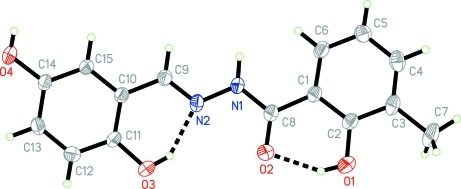
The molecular structure of the title compounds with atom labels and 30% probability displacement ellipsoids for non-H atoms. Intramolecular hydrogen bonds are shown as dashed lines.

**Fig. 2.**
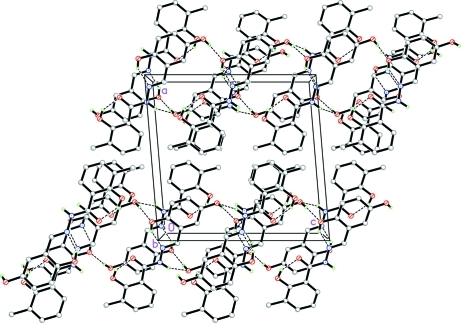
The molecular packing of the title compound as viewed down b. Hydrogen bonds are shown as dashed lines.

## supplementary crystallographic information

### Comment

Hydrazone compounds have been widely investigated for their biological properties (Patil *et al.*, 2010; Cukurovali *et al.*, 2006). Furthermore, the crystal structures of the hydrazone compounds have also attracted much attention in recent years (Mohd Lair *et al.*, 2009; Lin & Sang, 2009; Suleiman Gwaram *et al.*, 2010). As a continuation of our work on the structural characterization of such compounds (Han & Zhao, 2010a,b), the title new hydrazone compound is reported.

In the title compound, Fig. 1, the dihedral angle between the two benzene rings is 4.1 (2)°. The molecule adopts an *E* configuration with respect to the C═N bond. There are intramolecular O–H···N and O–H···O hydrogen bonds in the molecule (Table 1). All the bond lengths are within normal ranges (Allen *et al.*, 1987), and are comparable with those in the similar compounds (Li & Ban, 2009; Lo & Ng, 2009; Ning & Xu, 2009; Zhu *et al.*, 2009).

In the crystal structure, molecules are linked through intermolecular N–H···O and O–H···O hydrogen bonds (Table 1) to form chains running along the *c* axis (Fig. 2).

### Experimental

A mixture of 2,5-dihydroxybenzaldehyde (0.138 g, 1 mmol) and 2-hydroxy-3-methylbenzohydrazide (0.166 g, 1 mmol) in 50 ml methanol was stirred at room temperature for 1 h. The mixture was filtered to remove impurities, and then left at room temperature. After a few days, single crystals of the title compound, suitable for X-ray diffraction, were formed.

### Refinement

Amino H atom was located from a difference Fourier map and refined isotropically, with N–H distance restrained to 0.90 (1) Å. Other H atoms were positioned geometrically and refined using the riding-model approximation, with C–H = 0.93 or 0.96 Å, O–H = 0.82 Å, and U_iso_(H) = 1.2U_eq_(C) or U_iso_(H) = 1.5U_eq_(methyl C and O).

### Crystal data

**Table:**

C_15_H_14_N_2_O_4_	*F*(000) = 600
*M**_r_* = 286.28	*D*_x_ = 1.424 Mg m^−^^3^
Monoclinic, *P*2_1_/*c*	Mo *K*α radiation, λ = 0.71073 Å
*a* = 13.333 (2) Å	Cell parameters from 1316 reflections
*b* = 7.316 (1) Å	θ = 2.7–24.5°
*c* = 13.738 (2) Å	µ = 0.11 mm^−^^1^
β = 94.742 (2)°	*T* = 298 K
*V* = 1335.5 (3) Å^3^	Block, colorless
*Z* = 4	0.25 × 0.23 × 0.22 mm

### Data collection

**Table:**

Bruker SMART CCD area-detector diffractometer	3002 independent reflections
Radiation source: fine-focus sealed tube	1457 reflections with *I* > 2σ(*I*)
graphite	*R*_int_ = 0.087
ω scans	θ_max_ = 27.5°, θ_min_ = 1.5°
Absorption correction: multi-scan (*SADABS*; Bruker, 2001)	*h* = −17→16
*T*_min_ = 0.974, *T*_max_ = 0.977	*k* = −9→9
7636 measured reflections	*l* = −11→17

### Refinement

**Table:**

Refinement on *F*^2^	Primary atom site location: structure-invariant direct methods
Least-squares matrix: full	Secondary atom site location: difference Fourier map
*R*[*F*^2^ > 2σ(*F*^2^)] = 0.052	Hydrogen site location: inferred from neighbouring sites
*wR*(*F*^2^) = 0.136	H atoms treated by a mixture of independent and constrained refinement
*S* = 0.82	*w* = 1/[σ^2^(*F*_o_^2^) + (0.0615*P*)^2^] where *P* = (*F*_o_^2^ + 2*F*_c_^2^)/3
3002 reflections	(Δ/σ)_max_ = 0.001
197 parameters	Δρ_max_ = 0.22 e Å^−^^3^
1 restraint	Δρ_min_ = −0.26 e Å^−^^3^

### Special details

**Table:**

Geometry. All esds (except the esd in the dihedral angle between two l.s. planes) are estimated using the full covariance matrix. The cell esds are taken into account individually in the estimation of esds in distances, angles and torsion angles; correlations between esds in cell parameters are only used when they are defined by crystal symmetry. An approximate (isotropic) treatment of cell esds is used for estimating esds involving l.s. planes.
Refinement. Refinement of *F*^2^ against ALL reflections. The weighted *R*-factor wR and goodness of fit *S* are based on *F*^2^, conventional *R*-factors *R* are based on *F*, with *F* set to zero for negative *F*^2^. The threshold expression of *F*^2^ > σ(*F*^2^) is used only for calculating *R*-factors(gt) etc. and is not relevant to the choice of reflections for refinement. *R*-factors based on *F*^2^ are statistically about twice as large as those based on *F*, and *R*- factors based on ALL data will be even larger.

### Fractional atomic coordinates and isotropic or equivalent isotropic displacement parameters (Å^2^)

**Table:**

	*x*	*y*	*z*	*U*_iso_*/*U*_eq_	
O1	0.25674 (12)	0.4205 (2)	0.31421 (10)	0.0660 (5)	
H1	0.2036	0.3785	0.2893	0.099*	
O2	0.12591 (10)	0.3298 (2)	0.17731 (10)	0.0530 (4)	
O3	−0.10549 (10)	0.1488 (2)	0.03610 (9)	0.0528 (4)	
H3	−0.0498	0.1958	0.0467	0.079*	
O4	−0.22237 (11)	0.1219 (2)	−0.35891 (9)	0.0555 (4)	
H4	−0.1937	0.2007	−0.3888	0.083*	
N1	0.15386 (12)	0.3685 (2)	0.01891 (11)	0.0451 (4)	
H1A	0.1913 (15)	0.405 (3)	−0.0290 (12)	0.080*	
N2	0.06052 (11)	0.2950 (2)	−0.00814 (11)	0.0441 (4)	
C1	0.28436 (13)	0.4534 (3)	0.14397 (13)	0.0401 (5)	
C2	0.31477 (14)	0.4737 (3)	0.24325 (14)	0.0447 (5)	
C3	0.40822 (15)	0.5532 (3)	0.27436 (15)	0.0513 (6)	
C4	0.46896 (16)	0.6081 (3)	0.20408 (17)	0.0573 (6)	
H4A	0.5310	0.6606	0.2235	0.069*	
C5	0.44147 (15)	0.5884 (3)	0.10597 (16)	0.0570 (6)	
H5	0.4846	0.6271	0.0602	0.068*	
C6	0.34987 (14)	0.5113 (3)	0.07559 (14)	0.0496 (5)	
H6	0.3315	0.4976	0.0092	0.060*	
C7	0.43835 (18)	0.5770 (4)	0.38160 (16)	0.0771 (8)	
H7A	0.5062	0.6219	0.3903	0.116*	
H7B	0.4342	0.4615	0.4142	0.116*	
H7C	0.3939	0.6628	0.4088	0.116*	
C8	0.18276 (14)	0.3793 (3)	0.11571 (14)	0.0412 (5)	
C9	0.03111 (14)	0.2882 (3)	−0.09837 (14)	0.0436 (5)	
H9	0.0720	0.3335	−0.1444	0.052*	
C10	−0.06605 (13)	0.2097 (3)	−0.12963 (13)	0.0385 (5)	
C11	−0.13067 (14)	0.1425 (3)	−0.06259 (13)	0.0404 (5)	
C12	−0.22281 (14)	0.0702 (3)	−0.09566 (15)	0.0470 (5)	
H12	−0.2653	0.0246	−0.0511	0.056*	
C13	−0.25242 (14)	0.0648 (3)	−0.19390 (14)	0.0462 (5)	
H13	−0.3150	0.0168	−0.2152	0.055*	
C14	−0.18970 (14)	0.1304 (3)	−0.26101 (13)	0.0413 (5)	
C15	−0.09706 (13)	0.2005 (3)	−0.22897 (13)	0.0418 (5)	
H15	−0.0544	0.2425	−0.2743	0.050*	

### Atomic displacement parameters (Å^2^)

**Table:**

	*U*^11^	*U*^22^	*U*^33^	*U*^12^	*U*^13^	*U*^23^
O1	0.0648 (10)	0.0952 (13)	0.0377 (8)	−0.0074 (9)	0.0018 (7)	0.0016 (8)
O2	0.0484 (8)	0.0677 (10)	0.0431 (8)	−0.0063 (7)	0.0044 (7)	0.0013 (7)
O3	0.0568 (9)	0.0703 (10)	0.0313 (8)	−0.0052 (8)	0.0034 (6)	0.0003 (7)
O4	0.0528 (9)	0.0748 (12)	0.0365 (8)	−0.0068 (7)	−0.0098 (7)	−0.0039 (7)
N1	0.0382 (9)	0.0598 (12)	0.0362 (10)	−0.0042 (8)	−0.0040 (7)	−0.0021 (8)
N2	0.0398 (9)	0.0505 (11)	0.0403 (10)	−0.0002 (7)	−0.0068 (7)	−0.0037 (8)
C1	0.0392 (10)	0.0443 (12)	0.0356 (11)	0.0047 (9)	−0.0037 (9)	−0.0008 (9)
C2	0.0458 (11)	0.0502 (13)	0.0375 (11)	0.0054 (10)	−0.0001 (9)	0.0005 (9)
C3	0.0493 (12)	0.0555 (14)	0.0463 (13)	0.0090 (10)	−0.0138 (10)	−0.0075 (11)
C4	0.0376 (11)	0.0633 (16)	0.0685 (16)	0.0023 (10)	−0.0098 (11)	−0.0054 (12)
C5	0.0404 (12)	0.0762 (16)	0.0538 (14)	−0.0035 (11)	0.0004 (10)	0.0049 (12)
C6	0.0447 (11)	0.0667 (15)	0.0364 (11)	0.0021 (11)	−0.0031 (9)	−0.0001 (10)
C7	0.0754 (16)	0.098 (2)	0.0525 (15)	0.0085 (14)	−0.0247 (13)	−0.0169 (14)
C8	0.0413 (11)	0.0435 (12)	0.0378 (11)	0.0035 (9)	−0.0024 (9)	−0.0022 (9)
C9	0.0392 (11)	0.0523 (13)	0.0389 (12)	0.0015 (9)	0.0011 (9)	−0.0034 (10)
C10	0.0371 (10)	0.0439 (12)	0.0342 (11)	0.0028 (9)	0.0005 (8)	−0.0039 (9)
C11	0.0456 (11)	0.0445 (12)	0.0305 (11)	0.0037 (9)	−0.0006 (9)	−0.0016 (9)
C12	0.0446 (11)	0.0514 (13)	0.0456 (12)	−0.0002 (9)	0.0082 (10)	0.0012 (10)
C13	0.0354 (10)	0.0516 (13)	0.0505 (13)	−0.0042 (9)	−0.0023 (9)	−0.0051 (10)
C14	0.0399 (11)	0.0492 (12)	0.0337 (11)	0.0042 (9)	−0.0039 (9)	−0.0035 (9)
C15	0.0381 (11)	0.0549 (13)	0.0323 (11)	0.0000 (9)	0.0024 (8)	−0.0023 (9)

### Geometric parameters (Å, °)

**Table:**

O1—C2	1.351 (2)	C4—H4A	0.9300
O1—H1	0.8200	C5—C6	1.378 (3)
O2—C8	1.236 (2)	C5—H5	0.9300
O3—C11	1.370 (2)	C6—H6	0.9300
O3—H3	0.8200	C7—H7A	0.9600
O4—C14	1.380 (2)	C7—H7B	0.9600
O4—H4	0.8200	C7—H7C	0.9600
N1—C8	1.356 (2)	C9—C10	1.449 (3)
N1—N2	1.378 (2)	C9—H9	0.9300
N1—H1A	0.899 (19)	C10—C15	1.395 (2)
N2—C9	1.270 (2)	C10—C11	1.401 (3)
C1—C2	1.398 (2)	C11—C12	1.379 (3)
C1—C6	1.400 (3)	C12—C13	1.375 (3)
C1—C8	1.481 (3)	C12—H12	0.9300
C2—C3	1.409 (3)	C13—C14	1.381 (3)
C3—C4	1.371 (3)	C13—H13	0.9300
C3—C7	1.505 (3)	C14—C15	1.376 (2)
C4—C5	1.375 (3)	C15—H15	0.9300
			
C2—O1—H1	109.5	C3—C7—H7C	109.5
C11—O3—H3	109.5	H7A—C7—H7C	109.5
C14—O4—H4	109.5	H7B—C7—H7C	109.5
C8—N1—N2	117.67 (16)	O2—C8—N1	120.92 (18)
C8—N1—H1A	124.8 (15)	O2—C8—C1	121.82 (17)
N2—N1—H1A	117.5 (15)	N1—C8—C1	117.26 (18)
C9—N2—N1	118.55 (16)	N2—C9—C10	120.09 (18)
C2—C1—C6	118.40 (17)	N2—C9—H9	120.0
C2—C1—C8	118.67 (18)	C10—C9—H9	120.0
C6—C1—C8	122.87 (17)	C15—C10—C11	118.53 (17)
O1—C2—C1	122.42 (18)	C15—C10—C9	119.64 (18)
O1—C2—C3	116.41 (18)	C11—C10—C9	121.83 (17)
C1—C2—C3	121.17 (19)	O3—C11—C12	118.53 (18)
C4—C3—C2	117.80 (18)	O3—C11—C10	121.64 (17)
C4—C3—C7	122.02 (19)	C12—C11—C10	119.82 (17)
C2—C3—C7	120.2 (2)	C13—C12—C11	120.65 (19)
C3—C4—C5	122.33 (19)	C13—C12—H12	119.7
C3—C4—H4A	118.8	C11—C12—H12	119.7
C5—C4—H4A	118.8	C12—C13—C14	120.31 (17)
C4—C5—C6	119.8 (2)	C12—C13—H13	119.8
C4—C5—H5	120.1	C14—C13—H13	119.8
C6—C5—H5	120.1	C15—C14—O4	122.08 (18)
C5—C6—C1	120.46 (19)	C15—C14—C13	119.55 (17)
C5—C6—H6	119.8	O4—C14—C13	118.36 (16)
C1—C6—H6	119.8	C14—C15—C10	121.11 (18)
C3—C7—H7A	109.5	C14—C15—H15	119.4
C3—C7—H7B	109.5	C10—C15—H15	119.4
H7A—C7—H7B	109.5		

### Hydrogen-bond geometry (Å, °)

**Table:**

*D*—H···*A*	*D*—H	H···*A*	*D*···*A*	*D*—H···*A*
O1—H1···O2	0.82	1.82	2.5452 (19)	147
O3—H3···N2	0.82	1.85	2.576 (2)	146
O4—H4···O3^i^	0.82	1.97	2.774 (2)	169
N1—H1A···O4^ii^	0.90 (2)	2.27 (2)	3.072 (2)	148 (2)

Symmetry codes: (i) *x*, −*y*+1/2, *z*−1/2; (ii) −*x*, *y*+1/2, −*z*−1/2.
